# ParticipACTION: Overview and introduction of baseline research on the "new" ParticipACTION

**DOI:** 10.1186/1479-5868-6-84

**Published:** 2009-12-09

**Authors:** Mark S Tremblay, Cora L Craig

**Affiliations:** 1 Healthy Active Living and Obesity Research Group (HALO), Children's Hospital of Eastern Ontario Research Institute, 401 Smyth Road, Ottawa, ON, K1H 8L1, Canada; 2 Canadian Fitness and Lifestyle Research Institute, 201-185 Somerset Street West, Ottawa, Ontario, K2P 0J2, Canada

## Abstract

**Background:**

This paper provides a brief overview of the Canadian physical activity communications and social marketing organization "ParticipACTION"; introduces the "new" ParticipACTION; describes the research process leading to the collection of baseline data on the new ParticipACTION; and outlines the accompanying series of papers in the supplement presenting the detailed baseline data.

**Methods:**

Information on ParticipACTION was gathered from close personal involvement with the organization, from interviews and meetings with key leaders of the organization, from published literature and from ParticipACTION archives. In 2001, after nearly 30 years of operation, ParticipACTION ceased operations because of inadequate funding. In February 2007 the organization was officially resurrected and the launch of the first mass media campaign of the "new" ParticipACTION occurred in October 2007. The six-year absence of ParticipACTION, or any equivalent substitute, provided a unique opportunity to examine the impact of a national physical activity social marketing organization on important individual and organizational level indicators of success. A rapid response research team was established in January 2007 to exploit this natural intervention research opportunity.

**Results:**

The research team was successful in obtaining funding through the new Canadian Institutes of Health Research Intervention Research (Healthy Living and Chronic Disease Prevention) Funding Program. Data were collected on individuals and organizations prior to the complete implementation of the first mass media campaign of the new ParticipACTION.

**Conclusion:**

Rapid response research and funding mechanisms facilitated the collection of baseline information on the new ParticipACTION. These data will allow for comprehensive assessments of future initiatives of ParticipACTION.

## Background

### Out with the old and in with the new

ParticipACTION was launched as a physical activity communications and social marketing organization in 1971 with financial support from the Government of Canada [1-3]. The organization was strategically established outside of government as a private, not-for-profit and charitable corporation, allowing the corporation to work closely with and support government priorities while operating as a "business" in a lean, efficient, flexible and responsive manner. The corporation was able to generate substantial support from many partners including corporate, media, professional, volunteer and community groups, plus other governments [1-3].

During its 30-year mandate, ParticipACTION became a catalyst for physical activity across Canada and was viewed internationally as an exemplar of a highly successful national physical activity social marketing organization, with many award winning campaigns (for campaign examples see [3]). A number of studies have examined the influence of ParticipACTION in Canada and have addressed many aspects of its legacy including: its influence on future initiatives [3]; its effect on the physical activity movement in Canada [4]; the people behind the movement [5]; key aspects of the program's success [6]; its social marketing approach [2]; and, the challenge of running such a program in two official languages [7]. Details of the structure, function and campaigns of the original ParticipACTION are available elsewhere [8]. ParticipACTION's campaigns and initiatives have been informed by different theoretical frameworks over the years (as theoretical frameworks emerged).

After 30 years of continuous service, ParticipACTION ceased operations in 2001 due to funding cuts [9]. Between 2001 and 2007 no alternate organization surfaced to provide coordinated, sustained national physical activity promotion campaigns or broader social marketing, though many organizations and initiatives launched campaigns of varying reach and sustainability. In February 2007, after a six-year absence, and informed by a federal government commissioned feasibility study, Sport Canada and the Public Health Agency of Canada announced funding for the resurrection and revitalization of a "new" ParticipACTION. The mission of the new ParticipACTION is to provide leadership in collaborations and communications to foster the "movement" that inspires and supports Canadians to move more. To achieve this, ParticipACTION aspires to be a leading national catalyst for physical activity, engaging all Canadians in improving their health and the healthy nature of the communities in which they live, study, work and play, through physical activity and sport participation communications; encouraging and supporting the coordinated actions of many partner organizations; and contributing to community capacity building. The focus of the new ParticipACTION is not on physical activity program delivery, but rather social marketing, communications and partnership synergy [10]. The overall operation of the new ParticipACTION is guided by an ecological framework with individual campaigns informed by various frameworks and research suggesting promising practice.

During its first year of operations, ParticipACTION focused on developing and launching the first in a series of annual mass media campaigns. In subsequent years, it is planned to expand activities to include knowledge transfer; ongoing and coordinated communication; and supportive activities to increase the capacity of Canada's physical activity and sport delivery system, comprised of governmental and non-governmental organizations at the national and provincial/territorial level whose primary mandate is physical activity (for examples see http://www.activeliving.ca and http://www.sportmatters.ca) or who have a key interest in physical activity promotion (e.g. Chronic Disease Prevention Alliance of Canada, http://www.cdpac.ca) as well as locally based alliances to increase physical activity (e.g. Saskatoon in Motion, http://www.in-motion.ca). The mandate of the new ParticipACTION is contextualized within the broader *Integrated Pan-Canadian Healthy Living Strategy *[11] and the *Pan-Canadian Physical Activity Strategy *[12], each of which is yet to be resourced or implemented in a comprehensive fashion.

The new ParticipACTION's first mass media campaign was planned for an official launch in October 2007. ParticipACTION's campaign was designed to increase awareness of and create a sense of urgency about the low levels of physical activity among Canadian children. Four English and three French advertisements were developed to target parents of children 7-12 years. The advertising aired on a mix of conventional and specialty television broadcast channels from October 15, 2007 through March 31, 2008 and on radio from October 15 through December 31, 2007. Overall the advertisements were aired 8,963 times on television and 1,142 times on radio.

The six year hiatus in the operations of ParticipACTION provided an opportunity to assess a natural intervention (the re-emergence of the "new" ParticipACTION) by gathering individual-level data on awareness of the brand, campaign recall, knowledge, understanding and physical activity behaviours before and during the early period after the launch of the revitalized ParticipACTION. Baseline data were also collected at an organizational level to assess the future impact of a sustained campaign through ParticipACTION on the overall capacity of the physical activity sector in Canada.

### Capitalizing on an opportunity

During early discussions of the possible resurrection of ParticipACTION the lead author of this paper (MST) was appointed to the new Board of ParticipACTION and its Executive Committee. This privileged position allowed MST to facilitate strategic research planning extraneous to the operations of the Board and Executive. At the same time (early December 2006), though serendipitous to this strategic research planning, the Canadian Institutes of Health Research (CIHR) announced a new strategic funding opportunity titled the "Intervention Research (Healthy Living and Chronic Disease Prevention) Funding Program". This program was targeted towards funding research that examines "natural interventions" which are out of the control of researchers yet provide important, time-sensitive research opportunities. Accordingly, this funding opportunity did not follow the typical application and review process, allowing for applications to be submitted whenever the natural intervention opportunity presented itself (e.g. no fixed application date) and providing a thorough though expedited review process (for details on the funding program see http://www.researchnet-recherchenet.ca/rnr16/viewOpportunityDetails.do?prog=399&view=search&terms=intervention+research&org=CIHR&type=AND&resultCount=25). The combination of early knowledge of this ensuing natural intervention and the announcement of the CIHR funding program created a perfect opportunity to collect important baseline data before the official resurrection of ParticipACTION.

On January 20, 2007 a "rapid response research team" (RRRT) was convened in Saskatoon, Canada to discuss the appropriateness and viability of research possibilities to assess and study the impact of the revitalization and re-launch of ParticipACTION in the form of a natural intervention. The research team represented a good balance of experience with ParticipACTION, intervention research and natural experiments, and physical activity monitoring and measurement. The opportunity to collect pre-resurrection, baseline data at both individual and organizational levels was time-limited and required immediate action that this team could act upon. The objectives of this initial meeting were to:

• Take advantage of a natural experiment opportunity

• Test the new CIHR funding mechanism/opportunity

• Test the viability and responsiveness of the research community RRRT concept

• Link previously unlinked researchers and create networking opportunities

• Challenge ParticipACTION's commitment to research and evaluation

• Exploit and advance the learnings from the "Canada on the Move" initiative [13]

• Produce a letter of intent for the CIHR funding opportunity

Two letters of intent were generated at this initial meeting of the RRRT. One proposal focused on individual level data and a second on organizational level capacity (described further below). The cascade of events related to the preparation and outcome of this meeting are summarized below.

Key dates:

• CIHR funding announcement: December 8, 2006

• ParticipACTION resurrection funding confirmed: December 8, 2006

• Funding request to CIHR and ParticipACTION to host the initial RRRT meeting: December 19, 2006

• Notice of support for meeting funding request: December 22, 2006

• RRRT meeting: January 19-20, 2007

• Record of Discussion of meeting completed: January 21, 2007

• Two letters of intent submitted to CIHR: January 24, 2007

• Approval of two letters of intent received by email: February 21, 2007

• Two full research proposals submitted to CIHR: March 21, 2007

• Notification that both proposals were funded: June 18, 2007

• Data collection for both projects: August 2007 - February 2008

Six months passed between conception of the idea and full research funding being approved and within 13 months of the RRRT first meeting, the research data collection was complete. This very rapid sequence of events was necessary to collect baseline data before the first campaign of the "new" ParticipACTION was widely recognized.

### A success story

The ParticipACTION proposals were the first to be reviewed through the new CIHR funding mechanism and although the process went reasonably smoothly (as indicated by the sequence of events summarized above) the CIHR made subsequent adaptations to the review procedures in an effort to further expedite the process. In summary, the new CIHR funding opportunity allowed for the successful completion of this research - an opportunity previously unavailable through traditional funding mechanisms in Canada. Both the CIHR and the RRRT benefited and learned from this novel funding mechanism. This is a good example of constructive and productive cooperation and collaboration between researchers and a research granting agency.

### Overview of research and accompanying papers

To assess baseline awareness and understanding of ParticipACTION at an individual level, a population-based survey was conducted on a monthly basis over a period of six months from late August 2007 to February 2008. The survey consisted of a set of questions on the Physical Activity Monitor conducted by the Institute for Social Research at York University on behalf of the Canadian Fitness and Lifestyle Research Institute (CFLRI). The details of this project and the findings of the baseline data collection are described in the paper by Spence and colleagues [14].

The examination of population based physical activity campaigns has been limited with a focus on dissemination and accuracy, the behavioural effect of messages, and/or the recall of media items by the general public [15-17]. Less studied has been the impact such campaigns have on the broader organizational climate to mobilize and advocate for physical activity. The purpose of the organizational level research was to assess the baseline awareness of organizations regarding ParticipACTION, and their capacity to mobilize and advocate for physical activity. Using an internet-based survey instrument, provincial and national organizations from a range of sectors (e.g., sport, recreation, public health, education) provided responses for a quantitative assessment of organizational awareness of ParticipACTION and their capacity for physical activity promotion. The findings from the quantitative analysis of these data are presented in the paper by Plotnikoff et al. [18].

A sample of respondents from the quantitative survey [18] were contacted to particpate in a follow-up telephone interview to examine in more detail their organizational capacity for physical activity promotion, and the related barriers and facilitators, using qualitative research techniques. This rich baseline information on organizational capacity for physical activity promotion in a sample of Canadian organizations is summarized in the paper by Faulkner et al. [19].

An assessment of the inaugural mass communications campaign of the "new" ParticipACTION was completed using an internet-based survey conducted by Angus Reid Strategies. This survey evaluated awareness of ParticipACTION, its recent campaign and assessed the recall and interpretation of the campaign messaging in both the target market (parents with children aged 7-12 years) and others. The findings from this campaign evaluation are presented in the paper by Craig and co-workers [20].

Finally, Bauman et al. [21] provide a summary and commentary on the empirical papers contained in this supplement. This final paper helps by placing this research within the context of international initiatives to develop, implement, and evaluate mass media campaigns to promote physical activity.

## Conclusion

ParticipACTION has a distinguished history and can serve to inform future physical activity social marketing initiatives. The resurrection of ParticipACTION provided a unique research opportunity to establish baseline data to allow for future campaign assessments. A rapid response research team was established to exploit this opportunity and the new research funding opportunity of the CIHR allowed this opportunity to flourish. The findings from the baseline research are summarized in the papers that follow this brief introduction. This research provides a valuable platform for monitoring the future impact of ParticipACTION on the physical activity levels of Canadians. It is also hoped that this short series of papers informs the efforts of others attempting to assess the impact of physical activity communications and social marketing programs and campaigns elsewhere.

## Competing interests

The authors have been involved on various committees and advisory groups for both the previous and "new" ParticipACTION including the Board of Directors (MST). The Canadian Fitness and Lifestyle Research Institute (CFLRI) was previously contracted by the "new" ParticipACTION to perform research and surveillance - CLC is President of CFLRI but received no personal compensation for the contracted work.

## Authors' contributions

MST conceived of and initiated the study of the natural intervention of the re-emergence of the "new" ParticipACTION. MST and CLC co-wrote this manuscript.

## References

[B1] de Gaspé Beaubien P Foreword Can J Public Health 2004 95 Suppl 2 S4

[B2] Edwards P No country mouse: thirty years of effective marketing and health communications Can J Public Health 2004 95 Suppl 2 S6 S13 15250599 10.1007/BF03403997 PMC6976097

[B3] Rootman I Edwards P The best laid schemes of mice and men... ParticipACTION's legacy and the future of physical activity promotion in Canada Can J Public Health 2004 95 Suppl 2 S37 S44 15250605 10.1007/BF03404003 PMC6975637

[B4] Bauman A Madill J Craig CL Salmon A ParticipACTION: this mouse roared, but did it get the cheese? Can J Public Health 2004 95 Suppl 2 S14 S19 15250600 10.1007/BF03403998 PMC6975787

[B5] Edwards P The Mouseketeers^®^: people make the difference Can J Public Health 2004 95 Suppl 2 S33 S36 15250604 10.1007/BF03404002 PMC6975646

[B6] Lagarde F The mouse under the microscope: keys to ParticipACTION's success Can J Public Health 2004 95 Suppl 2 S20 S24 15250601 10.1007/BF03403999 PMC6975799

[B7] Lagarde F The challenge of bilingualism: ParticipACTION campaigns succeeded in two languages Can J Public Health 2004 95 Suppl 2 S30 S32 15250603 10.1007/BF03404001 PMC6975764

[B8] The ParticipACTION Archive Project http://www.usask.ca/archives/participaction/english/home.html

[B9] Knox M Foreword Can J Public Health 2004 95 Suppl 2 S5

[B10] ParticipACTION http://www.participaction.com/en-us/AboutParticipaction/AboutParticipaction.aspx

[B11] Secretariat for the Intersectoral Healthy Living Network, F/P/T Healthy Living Task Group, F/P/T Advisory Committee on Population Health and Health Security The Integrated Pan-Canadian Healthy Living Strategy 2005 Cat. No. HP10-1/2005. Minister of Health 2005 http://www.prontario.org/PDF/Healthy_Living_Strategy-ENG1.pdf

[B12] Coalition for Active Living The Pan-Canadian Physical Activity Strategy Coalition for Active Living 2005 http://www.activeliving.ca/English/index.cfm?fa=WhatWeDo.main

[B13] Craig CL Cragg SE Tudor-Locke C Bauman A Proximal impact of Canada on the Move Can J Public Health 2006 97 Suppl S21 S27 16676835 10.1007/BF03405361 PMC6975740

[B14] Spence JC Brawley LR Craig CL Plotnikoff RC Tremblay MS Bauman A Faulkner GEJ Chad K Clark MI ParticipACTION: Awareness of the participACTION campaign among Canadian adults - Examining the knowledge gap hypothesis and a hierarchy-of-effects model Int J Behav Nutr Phys Act 2009 6 85 10.1186/1479-5868-6-85 19995456 PMC2795738

[B15] Finlay SJ Faulkner G Physical activity promotion through the mass media: inception, production, transmission and consumption Prev Med 2005 40 121 30 10.1016/j.ypmed.2004.04.018 15533520

[B16] Hillsdon M Cavill N Nanchahal K Diamond A White IR National level promotion of physical activity: results from England's ACTIVE for LIFE campaign J Epidemiol Community Health 2001 55 755 61 10.1136/jech.55.10.755 11553661 PMC1731780

[B17] Bauman A Smith BJ Maibach EW Reger-Nash B Evaluation of mass media campaigns for physical activity Evaluation and Program Planning 2006 29 312 322 10.1016/j.evalprogplan.2005.12.004

[B18] Plotnikoff RC Todosijczuk I Faulkner G Pickering MA Cragg S Chad K Spence JC Tremblay M Craig CL Bauman A Brawley L Gauvin L ParticipACTION: Baseline assessment of the 'new ParticipACTION': A quantitative survey of Canadian organizational awareness and capacity Int J Behav Nutr Phys Act 2009 6 86 10.1186/1479-5868-6-86 19995457 PMC2799383

[B19] Faulkner G McCloy C Plotnikoff RC Bauman A Brawley LR Chad K Gauvin L Spence JC Tremblay MS ParticipACTION: Baseline assessment of the capacity available to the 'New ParticipACTION': A qualitative study of Canadian organizations Int J Behav Nutr Phys Act 2009 6 87 10.1186/1479-5868-6-87 19995458 PMC2796988

[B20] Craig CL Bauman A Gauvin L Robertson J Murumets K ParticipACTION: A mass media campaign targeting parents of inactive children; knowledge, saliency, and trialing behaviours Int J Behav Nutr Phys Act 2009 6 88 10.1186/1479-5868-6-88 19995459 PMC2797491

[B21] Bauman A Cavill N Brawley L ParticipACTION: the future challenges for physical activity promotion in Canada Int J Behav Nutr Phys Act 2009 6 89 10.1186/1479-5868-6-89 19995460 PMC2797762

